# Infrared thermography as a technique to measure physiological stress in birds: Body region and image angle matter

**DOI:** 10.14814/phy2.14865

**Published:** 2021-05-31

**Authors:** Joshua K. R. Tabh, Gary Burness, Oliver H. Wearing, Glenn J. Tattersall, Gabriela F. Mastromonaco

**Affiliations:** ^1^ Environmental and Life Sciences Graduate Program Trent University Peterborough ON Canada; ^2^ Department of Wildlife and Science Toronto Zoo Scarborough ON Canada; ^3^ Department of Biology Trent University Peterborough ON Canada; ^4^ Department of Biology McMaster University Hamilton ON Canada; ^5^ Department of Biological Sciences Brock University St Catharines ON Canada

**Keywords:** autonomic nervous system, infrared thermography, stress, thermoregulation

## Abstract

In vertebrates, changes in surface temperature following exposure to an acute stressor are thought to be promising indicators of the physiological stress response that may be captured noninvasively by infrared thermography. However, the efficacy of using stress‐induced changes in surface temperature as indicators of physiological stress‐responsiveness requires: (1) an understanding of how such responses vary across the body, (2) a magnitude of local, stress‐induced thermal responses that is large enough to discriminate and quantify differences among individuals with conventional technologies, and (3) knowledge of how susceptible measurements across different body regions are to systematic error. In birds, temperature of the bare tissues surrounding the eye (the periorbital, or “eye,” region) and covering the bill have each been speculated as possible predictors of stress physiological state. Using the domestic pigeon (*Columba* *livia domestica*; *n* = 9), we show that stress‐induced changes in surface temperature are most pronounced at the bill and that thermal responses at only the bill have sufficient resolution to detect and quantify differences in responsiveness among individuals. More importantly, we show that surface temperature estimates at the eye region experience greater error due to changes in bird orientation than those at the bill. Such error concealed detection of stress‐induced thermal responses at the eye region. Our results highlight that: (1) in some species, bill temperature may serve as a more robust indicator of autonomic stress‐responsiveness than eye region temperature, and (2) future studies should account for spatial orientation of study individuals if inference is to be drawn from infrared thermographic images.

## Graphical abstract

In vertebrates, changes in surface temperature following exposure to an acute stressor are thought to be promising indicators of the physiological stress response that may be captured non‐invasively by infrared thermography. However, the efficacy of using changes in surface temperature as indicators of physiological stress‐responsiveness requires: (1) an understanding of how such responses vary across the body (2) a magnitude of local, stress‐induced thermal responses that is large enough to discriminate and quantify differences among individuals with conventional technologies, and (3) knowledge of how susceptible measurements across different body regions are to systematic error. Using the Domestic Pigeon (*Columba* *livia domestica*; *n* = 9), we show that both the capacity to detect and quantify surface temperature responses to stress‐exposure among individuals depends upon the body region of observation and whether common sources of measurement error (here, individual orientation) are controlled.

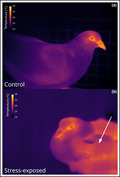

## 1 INTRODUCTION

An influence of acute stress exposure on body temperature in humans has been known to the medical community for centuries (Yeo, 2005). Over the past 50 years, observations of stress‐induced changes in body temperature across various vertebrates (reviewed elsewhere; Oka et al., 2001) have suggested that this phenomenon may be exploited to approximate stress‐responsiveness in both captive and free‐living species (i.e., using noninvasive technologies such as infrared thermography; Jerem et al., 2015; Herborn et al., 2015). The efficacy of doing so, however, requires an understanding of the physiological mechanisms controlling stress‐induced thermal responses (both locally and at the level of the brain), knowledge of how such responses may differ across anatomical regions of the body, and an awareness of how susceptible measurements across different anatomical regions may be to systematic error. Without such knowledge, the capacity to both infer and quantify aspects of stress‐physiological function from simple measurements of stress‐induced changes in body temperature remains limited.

To date, thermal responses to acute stress exposure are thought to arise from regional changes in both vascular flow and nonshivering thermogenesis (Oka et al., 2001; Shibata & Nagasaka, 1982)—each of which are largely mediated by the autonomic nervous system (“ANS”; i.e., by sympathetic activation or parasympathetic withdrawal; Nakamori et al., 1993; Oka et al., 2001). As such, the magnitudes of stress‐induced changes in body temperature (particularly at the body surface) have been speculated as indirect measures of ANS‐mediated stress responsiveness among individuals (Herborn et al., 2015; Jerem et al., 2015). The validity of this speculation, however, hinges upon two basic assumptions being met: (1) that ANS control over temperature of the observed body region is known, and (2) that the resolution of regional, stress‐induced thermal responses are large enough to discriminate and meaningfully quantify differences among individuals with conventional technologies (e.g., infrared thermographic cameras). In birds, temperature fluctuations at the richly vascularized region surrounding the eye (e.g., the periorbital region, henceforth, the “eye region”) have been proposed as useful metrics of physiological stress‐responsiveness (Edgar et al., 2013; Herborn et al., 2015; Jerem et al., 2015; Robertson et al., 2020b), with ANS‐mediation of local vascular flow (i.e., via constriction of the ophthalmic artery and rete ophthalmicum bypass arterioles) being described in some species (Cuthbertson et al., 1997; Midtgård, 1985). Intriguingly, several studies have reported correlations between circulating concentrations of corticosterone—a steroid hormone known to modulate stress‐induced ANS responsiveness (Sapolsky et al., 2000)—and eye region temperature in both captive and free‐living bird species (Jerem et al., 2018, 2019; Herborn et al., 2015; Ouyang et al., 2021; suggestive evidence in Herborn et al., 2018; but see Jerem, 2019). Such findings provide reasonable support for the first criteria of ANS control over stress‐induced changes in temperature at the eye region. Support for the second criteria at this region (i.e., a sufficient resolution to detect and quantify individual differences in stress‐induced thermal responsiveness), however, remains somewhat sparse. For example, while the magnitude of stress‐induced thermal responses at the eye region varies across species, most responses appear to be limited (e.g., 0.4–0.6°C in domestic Hens, *Gallus* *gallus*; Edgar et al., 2013; Herborn et al., 2015; approximately 1.0°C in Budgerigars, *Melopsittacus* *undulates*; Ikkatai & Watanabe, 2015; slightly below and within the accuracy of most thermographic cameras [±1–±4°C]). Given the close cerebral proximity of the eye region, and thus, high demand for local temperature regulation by counter‐current exchangers (e.g., the rete ophthalmicum, Midtgård, 1983), such limited responsiveness is arguably unsurprising. By contrast, bill temperature, being unconstrained by cerebral proximity and counter‐current vascular arrangement, displays remarkable thermal flexibility (>15°C in Pekin ducks, *Anas platyrhynchos*, Hagan & Heath, 1980; >10°C in great tits, *Parus* *major*, Winder et al., 2020) that is also thought to be mediated by ANS activity (discussed in Tattersall et al., 2017; see correlation between corticosterone and bill temperature in Weimer et al., 2020). Similar to the eye region, the bill is also uninsulated, highly vascularized (Tattersall et al., 2017), and typically visible to experimenters, rendering it a potentially valuable region to monitor rapid changes in temperature that may accompany changes in ANS function following an acute stress exposure. To our knowledge, however, the bill remains untested as an indicator of stress responsiveness (but see suggestive evidence in Weimer et al., 2020; Winder et al., 2020).

More practically, the relative efficacy of approximating ANS‐mediated stress responsiveness from measurements of eye region or bill temperature also depends upon how susceptible measurements from each anatomical region are to systematic biases. Recently, variation in the spatial orientation of individuals within infrared thermographic images has been raised as a possible source of error in surface temperature measurement (Playà‐Montmany & Tattersall, 2021; Winder et al., 2020). Specifically, changes in the angle of incidence of a focal object have been shown to alter its perceived emissivity, thus resulting in consistent under‐estimation of surface temperatures at certain angles (Playà‐Montmany & Tattersall, 2021) that may conceal or distort true changes in surface temperatures that are driven by biological functions (e.g., vasconstriction or vasodilation). Critically, the degree to which angle of incidence influences emissivity and surface temperature estimates appears to vary across biological tissues (Playà‐Montmany & Tattersall, 2021). As such, surface temperatures of some body regions may suffer from greater error due to variations in angle of incidence than others, with possible implications on the capacity to detect or quantify stress‐induced changes in body surface temperature across varying anatomical regions. In regions where stress‐induced changes in surface temperature are likely to be small (e.g., at the eye region; Edgar et al., 2013; Jerem et al., 2015; Herborn et al., 2015; Ikkatai & Watanabe, 2015; discussed above), such responses may be more susceptible to masking or distortion by errors attributed to variations in spatial orientation than at regions where stress‐induced changes in surface temperature are expected to be large (e.g., the bill; Hagan & Heath, 1980; Winder et al., 2020). To date, however, no studies have yet quantified stress‐induced changes in surface temperature while adequately controlling for individual orientation in 3‐dimensional space (but see early efforts in Herborn et al., 2018; Winder et al., 2020), nor have any studies tested whether and how the effects of spatial orientation may bias detection of such responses across anatomical regions.

Using the domestic pigeon (*Columba* *livia domestica*) as a model species, we tested whether surface temperature responses to an acute stressor vary across the facial region. In addition, we tested whether changes in head orientation differentially affected our capacity to detect stress‐induced thermal responses across facial regions, and whether the region at which stress‐induced thermal responses are measured influences the capacity to detect and quantify individual differences in stress‐responsiveness. We predicted: (1) that exposure to an acute stressor would elicit a larger thermal response at the bill when compared with the eye region, consistent with predicted differences in ANS‐mediated thermal flexibility of each region, (2) that increased thermal flexibility at the bill would ensure detection of stress‐induced thermal responses, despite measurement errors attributed to variation in head orientation, and (3) that increased thermal flexibility at the bill would permit greater discrimination of individual differences in stress‐responsiveness than would the eye region.

## 2 METHODS

All experimental methods described in this study were approved by the Trent University and Toronto Zoo Animal Care Committees (Animal Use Protocol #: 25060 and 2017‐10‐01, respectively).

### 2.1 Study sample

Adult domestic pigeons (*n*_female_ = 5, *n*_male_ = 5; body mass range: 326–470 g) were obtained from a local breeder in November, 2017. One month prior to experimentation, individuals were transferred to the Toronto Zoo for acclimation in separate, galvanized steel enclosures (0.75 m × 0.50 m × 0.35 m: length × width × height; 2.5 cm × 2.5 cm grid) with straw bedding. Enclosures were held in a common room within the Wildlife Health Center to permit vocal and visual interaction among individuals, and our common room was held at 40% humidity, 14:10 h (light:dark), and 18°C (below thermoneutrality, at which robust stress‐induced changes in surface temperature have been previously reported for other avian species Jerem et al., 2019; Nord & Folkow, 2019; lower critical temperature for Domestic Pigeons = 22°C; Calder & Schmidt‐Nielsen, 1967),  for the duration of the study (2 months). Temperature and humidity of our common room was centrally controlled and were unlikely to vary across the duration of a given experiment (approximately 15 min). Food (dried corn, milo, safflower seed, peas, sliced apple, romaine lettuce, and ground oyster shells) and water were provided *ad libitum* throughout acclimation and experimentation.

Following a 1 month acclimation, individuals were sequentially fitted with central venous catheters (left jugular) under isofluorane anesthesia to permit blood sampling during experimental procedures that are described below. All blood samples were, however, deemed insufficient for laboratory analysis. Data from blood sampling, therefore, are not discussed. All individuals were given a minimum of three days to recover from cannulation surgeries, during which they were provided intravenous meloxicam (2.0 mg/kg) for mitigation of any pain. Behavioral observations suggested a lack of discomfort among individuals following the three‐day recovery (e.g., regular feeding, movement, and vocalizing). Cannulas remained in place for all experimental procedures (again, described below) and previous avian studies suggest that cannula retention was unlikely to influence the magnitude of the physiological stress response in our study animals (Korte et al., 1997; Le Maho et al., 1992).

### 2.2 Experimental procedure and thermographic imaging

**FIGURE 1:**
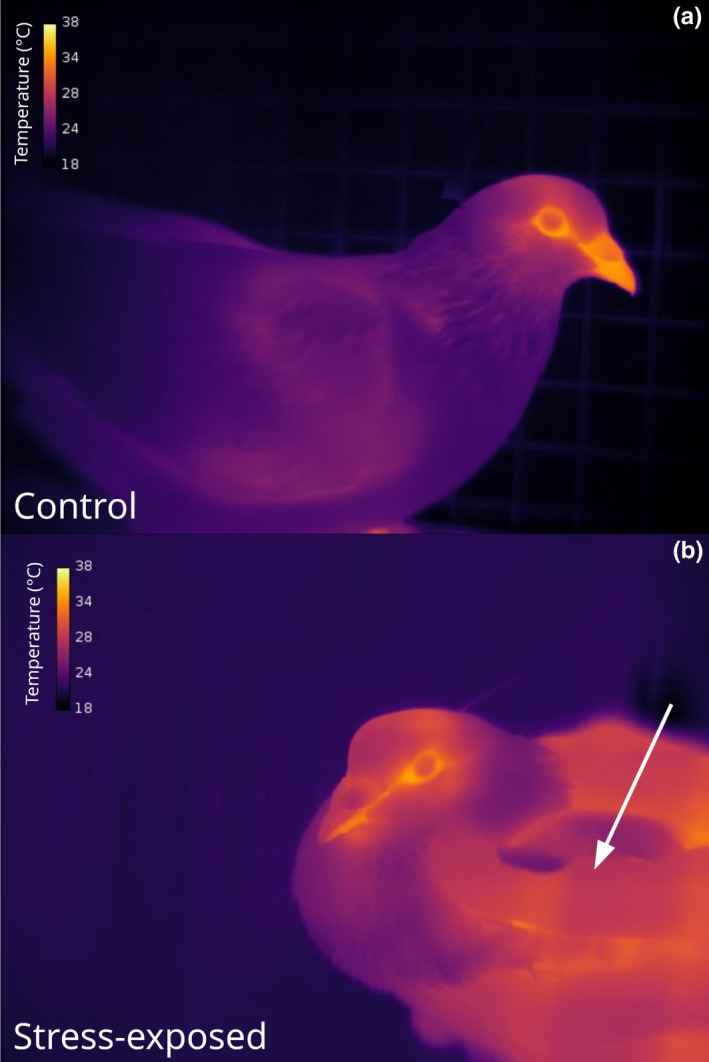
Infrared thermographic images of Domestic Pigeons collected during two separate experimental treatment types. Pixel coloration is scaled according to pixel temperature (°C). (a) Individual in control treatment. (b) Individual in acute stress exposure treatment. Hand of the experimenter is indicated by the white arrow

To monitor regional surface temperature responses to an acute stressor, we used infrared thermography under paired, experimental stress‐exposure and control conditions. Upon recovery from surgeries, pigeons were randomly assigned to a treatment type (i.e., stress‐exposure or control) and sequentially selected for use in experimentation (*n* ≤ 4 per day). Those selected for use were acclimated to thermographic camera presence (Jerem et al., 2015) by placing mock cameras in front of their enclosures (black acrylonitrile butadiene styrene, or “ABS” piping on a tripod; 0.5 m distance), one day prior to experimental treatments. On the day of experimentation, mock cameras were replaced with an infrared thermographic camera (SC660^TM^, FLIR; 640 × 480 resolution; accuracy = ±1°C), and individuals were left to acclimate for 1 h. Next, thermographic filming was initiated remotely (image frequency = 2 Hz; Figure 1) and individuals of both treatments were left undisturbed and blind to experimenter presence for 3.5 min. Immediately after, individuals assigned to stress‐exposure treatments were captured in an ungloved hand and held stationary within their enclosure (perpendicular to our thermographic camera) for 3.5 min, thus permitting us to capture both rapid, and long‐lasting responses to handling (Herborn et al., 2015; Jerem et al., 2015; similar to Nord & Folkow, 2019). Those assigned to control treatments were left undisturbed for an equivalent time‐period (3.5 min). For all handled individuals, latency to capture was less than 5 s, however, the beginning of our stress exposure (i.e., “time 0”) was assumed to be the time at which enclosures were opened to permit handling. Nonetheless, onset of the stress response in our handled individuals may have preceded enclosure opening, owing to uncontrolled experimenter noise.

Following completion of 3.5 min, thermographic filming was stopped and handled individuals were released. Our study, therefore, differs from previous and similar studies in that we did not monitor the surface temperatures of individuals following release or during recovery (e.g., Herborn et al., 2015; Nord & Folkow, 2019). Throughout both control and stress‐exposure treatments, blood samples (~100 μl; between 0.1%–0.2% of estimated total blood‐volume, Palomeque & Planas, 1978) were collected through the central venous catheters at 3.5, 4.5, and 7.5 min after onset of filming. Because blood samples were collected from both control and stress‐exposed individuals, surface temperature responses to each treatment type are unlikely to be explained by blood sampling alone.

In total, nine individuals were filmed in acute stress exposure treatments (*n*_female_ = 5, *n*_male_ = 4), and five individuals (n_female_ = 2, n_male_ = 3) were filmed in control treatments, with four individuals being filmed in both treatment types. Among individuals that experienced both acute stress exposure treatments and control treatments, the order in which treatments occurred were varied (*n* = 2 received acute stress exposure treatments first and control treatments second on separate days, and *n* = 2 received control treatments first and acute stress exposure treatments second, on separate days). Given that control treatments did not involve interaction with experimenters, however, we do not expect individuals that had first experienced an acute stress‐exposure treatment to elicit a learned, stress‐physiological responses during infrared thermographic imaging.

### 2.3 Estimation of surface temperature

Raw radiance values from all suitable thermographic images (see below) were converted to surface temperature readings in the software FIJI (https://imagej.net/Fiji) according to Planck's law, and according to methods and equations described elsewhere (Minkina & Dudzik, 2009; Tattersall, 2016; Tattersall et al., 2020). Here, emissivity (*ε*) of the eye region and bill were assumed to be 0.95 (Best & Fowler, 1981) and ambient temperature and relative humidity were assumed to be fixed at 18°C and 40%, respectively. Calibration constants for our thermographic camera were extracted using the software Exiftool (https://exiftool.org/). Maximum eye region and bill temperature were then manually sampled from reconstructed images using FIJI (eye region ≈ 2200 pixels/image; bill ≈ 2000 pixels/image). Because the precise locations of thermal responses within the eye region (i.e., the periocular region or the cornea) and bill (i.e., the upper or lower mandible) were beyond the scope of this study, we measured the maximum temperatures for the entire periorbital region and the entire bill. Maximum temperature of each region was selected in place of regional means to reduce error associated with incorrect object perimeter selection (Jerem et al., 2015), and only images where individuals were stationary were used for surface temperature extraction (Tattersall, 2016). Given that our acute stress exposure treatments involved handling of individuals, extraction of surface temperatures could not be conducted blindly to treatment allocation. In total, data from 8331 thermographic images were used for this study (control treatments: *n*_images_ = 2533, *n*_images_/individual = 507 ± SD = 124; stress‐exposure treatments: *n*_images_ = 5798, *n*_images_/individual = 644 ± SD = 456).

### 2.4 Estimation of head orientation among study individuals

Recent studies have shown that the emissivity and perceived surface temperature of an object can vary according to angle of incidence in an infrared thermographic image (Playà‐Montmany & Tattersall, 2021; Winder et al., 2020). As such, changes in the relative orientation of an object during infrared thermographic imaging may conceal or distort true changes in surface temperature that are driven by biological processes (e.g., vasomotion or contraction of skeletal muscle). In this study, we sought to estimate the degree to which stress‐induced changes in surface temperature at the eye region and bill may be concealed or distorted by changes in orientation of the head of domestic pigeons, using thermographic images derived from a randomly sampled subset of our study individuals (*n* = 7 individuals; *n* = 4 control trials; and *n* = 4 stress‐exposure trials, with one individual experiencing both treatments).

**FIGURE 2:**
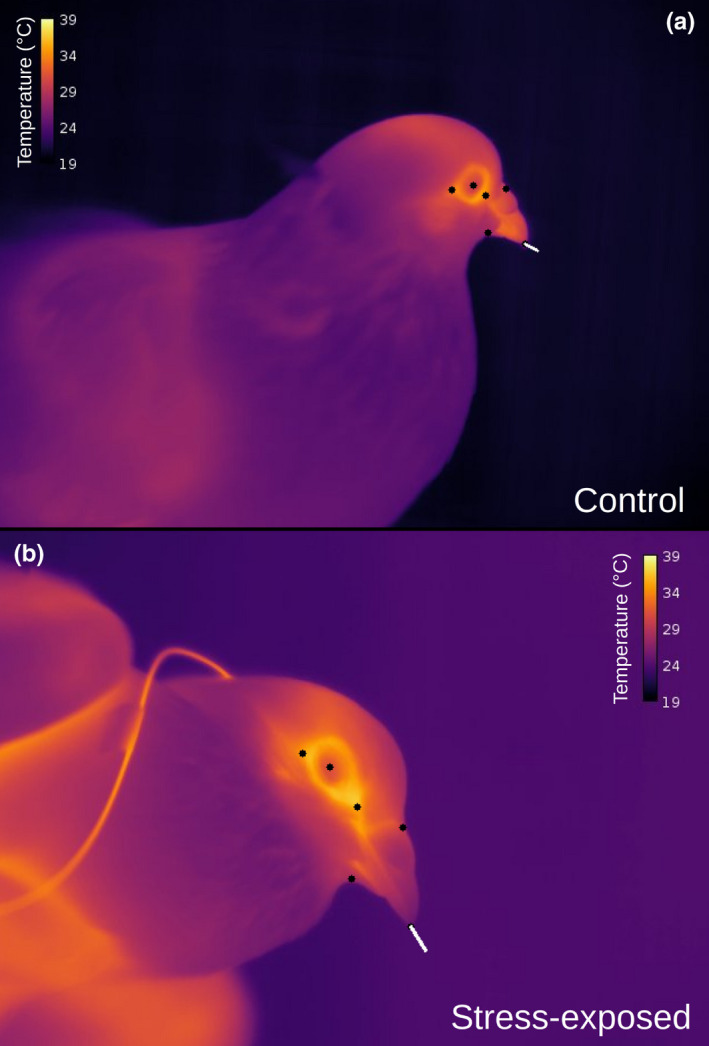
Representation of head orientation estimates drawn from landmarked thermographic images. Black dots represent landmarks and white lines are projected from the bill tip to a point approximately 1.0 cm directly anterior to the bill, according to orientation estimates drawn from position algorithms by Lepetit et al (2019). (a) Individual in control treatment. (b) Individual in acute stress exposure treatment

To estimate head orientation of sample individuals within thermographic images, we first determined the locations of 4–9 identifiable head regions (or “landmarks”; see Figure S1) of each individual across images using FIJI. Landmark locations included: the bill tip (a), the upper caudal cyr (b), the lower caudal mandible (c), the left and right lower rostral periorbital area (d, g), the center of the left and right eyeball (e, h), and the left and right lower caudal periorbital area (f, i; Figure S1). Next, the 2‐dimensional positions of each landmark (in pixels) were compared to those of landmarks drawn from a standardized digital image of a domestic pigeon (Figure S1), with locations in the 3‐dimensional world co‐ordinate system being estimated from morphological data on this species that is reported elsewhere (Donovan, 1978; Johnston, 1990; Goldberg, 1999; see Figure S1). Here, the goal of our comparisons were to calculate both a 3‐dimensional translation and a 3‐dimensional rotation of our imaged individual that was sufficient to explain the difference between the 2‐dimensional landmark positions of this individual, and the 2‐dimensional landmark positions of our standardized image (commonly known as the “perspective‐n‐point” problem; Haralick et al., 1994). To achieve this end, we loaded co‐ordinates of landmarks from both our imaged individuals and our standardized image into positional algorithms proposed by Lepetit et al (Lepetit et al., 2009), using the OpenCV library (https://pypi.org/project/opencv‐python) in Python (version 3.8.5; Python Software Foundation, 2021). Next, both translations (in pixels) and rotations (here, a relative pitch, yaw, and roll, in degrees) in 3‐dimensional space were extracted from algorithms (Figure 2). Because we were most interested in the effects of angle of incidence on estimates of eye region and bill surface temperature, however, only 3‐dimensional rotations were used in subsequent analyses.

To assess the accuracy of each translational and rotational estimate, we calculated the mean Euclidean distance between our true landmark positions and those predicted by our translation and rotation matrices (or “mean of the Euclidean residuals”) in pixels. Where the mean of our Euclidean residuals exceeded an arbitrary value of 25 (approximately ~1.0 cm at our image distance), estimates drawn from the associated matrices were excluded from our analyses (*n* = 82 estimates). In total, 3‐dimensional rotations were estimated from 4895 thermographic images across our randomly selected sample of seven individuals (mean images/individual ± SD = 612 ± 107).

### 2.5 Statistical analysis

All statistical analyses were conducted in R (version 3.6.1), and general additive mixed‐effects models (or “GAMMs”) were constructed in the R package “mgcv” (https://cran.r‐project.org/web/packages/mgcv/) with restricted maximum likelihood (*α* = 0.05). Models were validated by visually diagnosing residual distributions.

### 2.6 Effect of acute stress exposure on eye region and bill surface temperature

To test whether handling influenced eye region temperature in pigeons, we used a GAMM with maximum eye region temperature (°C; averaged within birds across 10 seconds to reduce temporal autocorrelation; *n* = 954 observations) as the response variable. Treatment (e.g., handling or control) was included as a fixed linear predictor, and time post‐experimental onset (henceforth “time” in seconds, wherein time “0” refers to the time at which enclosures were opened to permit captured in stress‐exposed treatments) was included as a nonlinear regression spline with three knots to capture a curvilinear relationship (Jerem et al., 2015) without over‐fitting. Here, knot positions were determined by truncated eigen decomposition (i.e., via use of a thin‐plate regression spline) and therefore reflect those that permitted our model to explain the most variance across our predicted nonlinear trend. To test whether treatment type influenced the relationship between surface temperature and time, an interaction (i.e., difference spline) between treatment and our time‐spline was also included. Finally, individual identity was included as a random intercept to control for repeated sampling among individuals. Remaining autocorrelation between adjacent temperature measurements was corrected using a type‐I autoregressive (“AR1”) covariance structure (*ρ* = 0.889; temporal autocorrelation in raw, unaveraged model: *ρ* = 0.946), and all data from one individual were excluded to correct for heteroskedasticity of residuals among individuals (Levene test: *F *= 6.055, *p* < 0.0001).

To test whether handling influenced bill temperature in our study individuals, we replicated our model described for eye region temperature but replaced the response variable with maximum bill temperature of our pigeons (°C; total surface temperature observations = 924). Bill temperature measurements displayed significant temporal auto‐correlation (bill temperature: *ρ* = 0.886; again, temporal autocorrelation in model with raw unaveraged data: *ρ* = 0.959). Our model was therefore fitted with an AR1 covariance structure.

### 2.7 Influence of head orientation on eye region and bill surface temperature estimates

First, we tested the effect of angle of incidence (here, the yaw of an individual's head relative to a standardized image; see “Estimation of individual orientation”) on the surface temperature of the eye region and bill, after controlling for effects of treatment type, time, and individual identity. To do so, we reconstructed our GAMMs described above, however, while only using surface temperature data obtained from individuals for which head orientation was successfully estimated. Effects of acute stress exposure on surface temperature trends across time differed little from those of our original models at an *α* of 0.05 (Table S1) despite a reduction in sample size. Next, the yaw of individuals at the time of thermographic image capture was included at a fixed and nonlinear covariate in our models using a thin‐plate regression split with five knots (nonlinear effects reported in Playà‐Montmany & Tattersall, 2021), and both models were subsequently re‐run. In our adjusted models, significant correlations between yaw and surface temperature values were expected to indicate systematic effects of angle of incidence on mean surface temperature values of a specific facial region.

To assess whether angle of incidence influenced the uncertainty of surface temperature estimates in our experiment, we tested whether the variance of eye region or bill temperature residuals (extracted from models described above) differed across the yaw of individuals at the time of image capture (i.e., displayed heteroskedasticity). To do so, we used two Breusch‐Pagan tests for heteroskedasticity in R. Finally, to analyze whether angle of incidence was sufficient to conceal or distort predicted changes in surface temperature following exposure to an acute stressor, we: (1) compared results of our GAMMs including yaw as a predictor (and a weighting factor, if heteroskedasticity was detected), and excluding yaw as a predictor, and (2) compared the likelihoods (here, log‐likelihood) of our GAMMs including yaw as a predictor, and excluding yaw as a predictor using two chi‐squared differences tests. Model likelihoods were compared to validate that changes in the significance levels of predictors between model iterations were likely to hold a meaningful influence on the capacity to explain region surface temperature values.

### 2.8 Variation in stress‐induced thermal responses at the eye region and bill among individuals

To analyze whether individual differences in stress‐induced thermal responses could be detected at the eye region or bill, we re‐ran our previously described GAMMs using data from all imaged individuals (i.e., regardless of whether spatial orientation was known) while allowing the relationship between time and regional temperature to vary across individuals during acute stress exposure treatments (here, by the inclusion of a random linear slope of the interaction between time and treatment). Next, we tested whether the inclusion of random slopes per individual improved the explanatory capacity of our models by comparing the log‐likelihoods of our original models with those of our individually adjusted models using two chi‐squared difference tests.

## 3 RESULTS

All means reported below are marginal ± standard errors of the means (SEM). Effects of all model covariates have therefore been averaged prior to the calculation of each mean.

### 3.1 Bill temperature but not eye region temperature declines after handling

**FIGURE 3:**
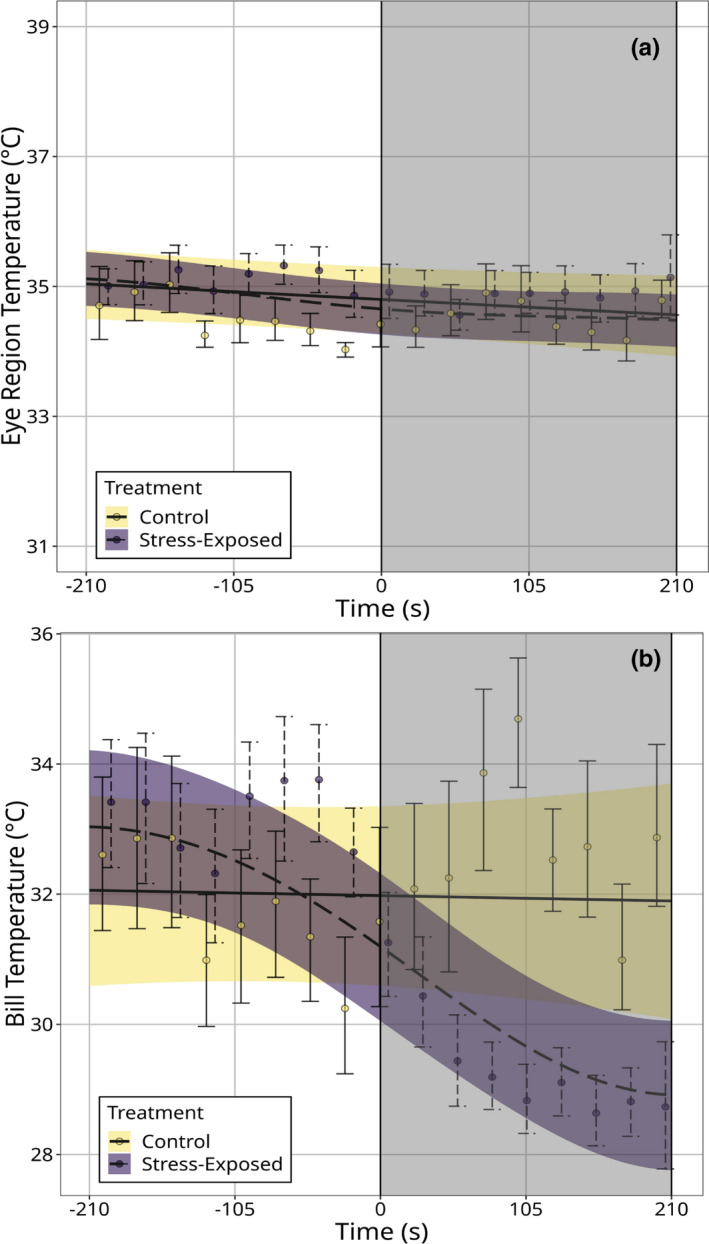
Surface temperature (°C) of Domestic Pigeons exposed to control and acute stress exposure (handling) treatments across time (seconds). Trends are estimated from *n* = 8331 infrared thermographic images. (a) Maximum eye region temperature (*n* = 9 individuals; *n*_control_ = 5, *n*_stress‐exposed_ = 8). (b) Maximum bill temperature (*n* = 10 individuals; *n*_control_ = 5, *n*_stress‐exposed_ = 9). Time 0 (vertical dashed lines) represents the time at which enclosures were opened to permit capture in acute stress exposure treatments, and grey boxes represent the time at which individuals were handled. Trend‐lines are estimated from generalized additive mixed‐effects models (“GAMMs”); ribbons represent 95% simultaneous confidence intervals around trends. Dots represent average surface temperature measurements across fifty seconds of observation, and across individuals (thus, the mean response across individuals). Error bars around dots represent 95% Wald confidence intervals around means. Bill temperature but not eye region temperature significantly declined after handling (*p*_eye region_ = 0.154: *p*_bill_ < 0.0001)

Eye region temperature was not significantly predicted by treatment type (*p* = 0.702; Table 1), time (*p* = 0.096; Table 1), or an interaction between each parameter (*p* = 0.154; Table 1; Figure 3A), suggesting that temperature of the eye region was unresponsive to handling. Indeed, mean eye region temperatures before and after handling were statistically indistinguishable (pre‐handling: 34.9°C ± 0.120; post‐handling: 34.5°C ± 0.116). Furthermore, eye region temperature during handling was highly similar to that of control individuals at equivalent times points (3.5–6.9 min; stress‐exposed: 34.5°C ± 0.116; control: 34.7°C ± 0.196). By contrast, while bill temperature was not significantly predicted by treatment type or time alone (*p*_treatment_ = 0.083, *p*_time_ = 0.864; Table 1), we detected a significant interaction between both parameters (*p* < 0.0001; Table 1), with bill temperature significantly declining in handling treatments but not control treatments (Figure 3B). Among individuals in acute stress exposure treatments, average bill temperature during handling was 2.6°C lower than that prior to handling (pre‐handling: 32.4°C ± 0.310; post‐handling: 29.8°C  ± 0.304), and when compared with control individuals at equivalent time periods, mean bill temperature during handling was 2.1°C lower in handled individuals (handled: 29.8°C  ± 0.304; controls: 31.9°C ± 0.577) than controls. Bill temperature did not return to baseline estimates before the termination of our experiment. Notably, neither baseline eye region temperature (marginal mean for the first 60 s of observation), nor baseline bill temperature significantly differed between treatment groups (eye region: $\overset{-}{x}$_controls_ = 35.0°C ± 0.173, $\overset{-}{x}$_stress‐exposed_ = 35.1°C ± 0.151, *p *= 0.688; bill: $\overset{-}{x}$_controls_ = 31.9°C ± 0.404, $\overset{-}{x}$_stress‐exposed_ = 31.2°C  ± 0.326, *p *= 0.190).

**TABLE 1:** Results of GAMMs testing the influence of acute stress exposure on facial surface temperature in Domestic Pigeons

Eye region temperature					
Parametric predictors					
Coefficient	Estimate (*β*)	SEM	*t*‐value	*p*‐value	
Intercept	34.756	0.271	128.350	<0.001*	
Treatment	−0.050	0.131	−0.382	0.702	
Smooth predictors					
Coefficient	Estimate (*β*)	SEM	e.d.f	*F*‐value	*p*‐value
Time	−0.066	0.060	1.002	2.776	0.096
Time:Treatment	0.073	0.130	0.882	1.003	0.154
Random predictors					
Coefficient	SEM				
Individual identity	0.349				

Bill temperature					
Parametric predictors					
Coefficient	Estimate (*β*)	SEM	*t*‐value	*p*‐value	
Intercept	31.557	0.690	45.760	<0.0001*	
Treatment	−0.589	0.339	−1.734	0.083	
Smooth predictors					
Coefficient	Estimate (*β*)	SEM	e.d.f	*F*‐value	*p*‐value
Time	−0.022	0.158	1.001	0.029	0.864
Time:Treatment	0.598	0.309	1.822	23.835	<0.001*
Random predictors					
Coefficient	SEM				
Individual identity	0.900				

Note

Effects of time (s), treatment (control or restraint), and individual identity are included. Estimates (*β*) and standard errors (SEM) of smooth terms are averaged across knots. Asterisks (*) denote significant effects. Eye region: *n* = 9 individuals, *n* = 947 observations; bill: *n* = 10 individuals, *n* = 924 observations. Deviance explained = 57.9% for GAMM predicting eye region temperature, and 60.8% for GAMM predicted bill temperature.

### 3.2 Angle of incidence influences mean eye region temperature estimates and masks stress‐induced temperature declines

Orientation of the head (“angle of incidence,” measured as yaw of the head) during thermographic imaging significantly predicted mean eye region temperature, but not mean bill temperature, after accounting for treatment type, time, and individual identity (eye region: *β* ±   SEM = 0.016  ±  0.006, *F* = 12.361, *p* < 0.001; bill; *β* ± SEM = −0.009 ± 0.016, *F* = 0.513, *p* = 0.474; Figure S2). Specifically, at the eye region alone, surface temperatures declined as angle of incidence increased, with estimates drawn at our minimum observed angle of incidence (35.060°C ± 0.080; yaw = −87.667°, where yaw = −90° represents orientation toward the thermographic camera) being approximately 0.4°C higher than those drawn at our maximum observed angle of incidence (34.647°C ± 0.068; yaw = 60.584°, where yaw = 90° represents an orientation away from the thermographic camera). Neither eye region temperature estimates nor bill temperature estimates were significantly heteroskedastic across angles of incidence (eye region: *X* ^2^ = 0.660, *p *= 0.417; bill: *X^2^* = 1.826, *p *= 0.177).

**FIGURE 4:**
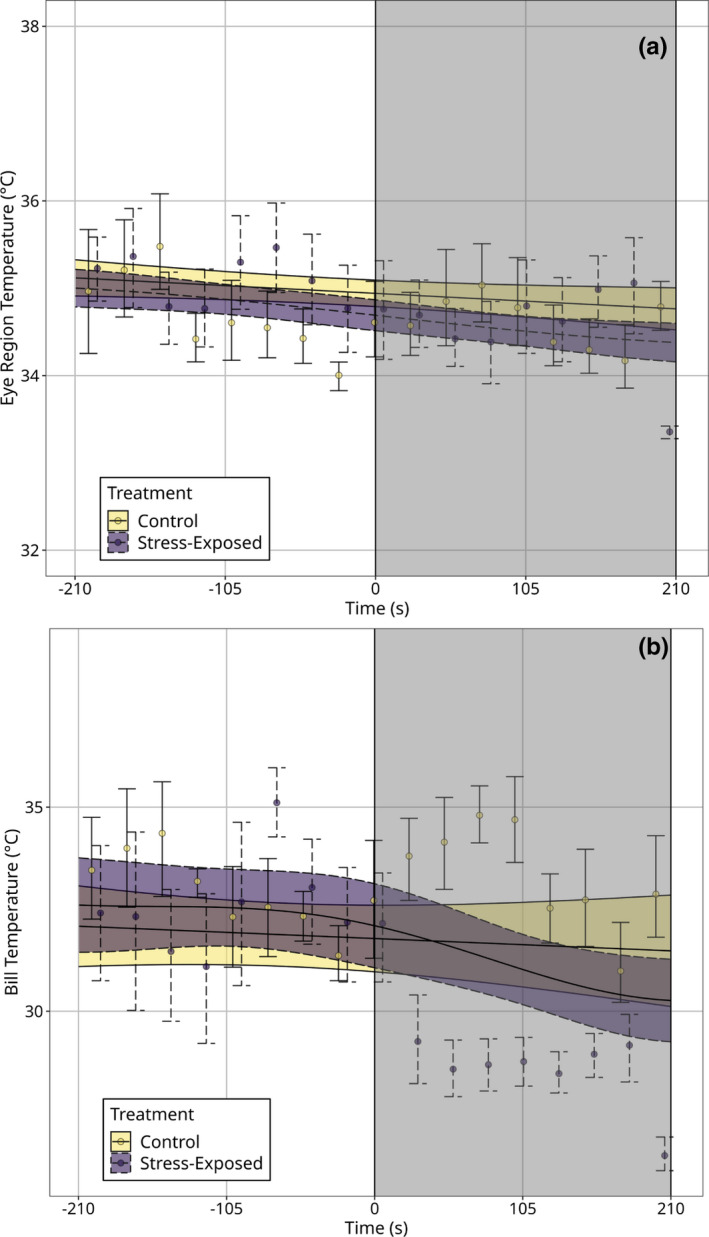
Effects of control and acute stress exposure treatments (handling) on surface temperature (°C) of Domestic Pigeons across time (seconds), after adjusting for effects of head orientation. Trends are estimated from *n* = 4895 infrared thermographic images captured from *n* = 7 individuals (*n* = 4 individuals per treatment, with on individual in both control and stress‐exposed treatments). (a) Effects of treatment type on maximum eye region temperature across times. (b) Effects of treatment type on maximum bill temperature across time. Time 0 (vertical dashed lines) represents the time at which enclosures were opened to permit capture in acute stress exposure treatments, and grey boxes represent the time at which individuals were handled. Trend‐lines are estimated from a generalized additive mixed‐effects model (“GAMM”); ribbons represent 95% Wald confidence intervals around trends. Dots represent average surface temperature measurements across ten seconds of observation, and across individuals. Errorbars around dots represent 95% Wald confidence intervals around means. Both bill and eye temperature significant decline in stress‐exposed (handled) treatments (*p*_eye region_ = 0.041: *p*_bill_ = 0.036)

In our model predicting mean eye region temperature, inclusion of angle of incidence as a covariate significantly improved log‐likehood estimates (*X* ^2^ = 10.230, *p* = 0.005). Furthermore, after including angle of incidence as a fixed predictor, a significant interaction between time and treatment type was detected (*p *= 0.041; Table 2), with eye region temperature significantly declining in stress‐exposed treatments (Figure 4a). Among stress‐exposed individuals, mean eye region temperature was approximately 0.4°C lower during handling than prior to handling (pre‐handling: 34.9°C ± 0.085; post‐handling: 34.5°C ± 0.085), and 0.4°C lower during handling than that of control individuals at equivalent time‐points (stress‐exposed: 34.5°C ± 0.085; control: 34.9°C ± 0.093). Time alone was also significantly correlated with mean eye region temperature (*p* = 0.037; Table 2), with mean eye region temperature declining by approximately 0.1°C across control treatments.

**TABLE 2:** Influence of angle of incidence and acute stress exposure on facial temperature of domestic pigeons; results of two GAMMs

Eye region temperature					
Parametric predictors					
Coefficient	Estimate (*β*)	SEM	*t*‐value	*p*‐value	
Intercept	34.831	0.404	86.297	<0.001*	
Treatment	−0.170	0.093	−1.825	0.069	
Smooth predictors					
Coefficient	Estimate (*β*)	SEM	e.d.f	*F*‐value	*p*‐value
Angle of incidence (Yaw)	−0.016	0.006	1.001	12.361	<0.001*
Time	−0.048	0.031	1.000	0.011	0.036*
Time:Treatment	0.045	0.077	1.065	22.533	0.041*
Random predictors					
Coefficient	SEM				
Individual identity	0.434				

Bill temperature					
Parametric predictors					
Coefficient	Estimate (*β*)	SEM	*t*‐value	*p*‐value	
Intercept	31.852	1.063	29.960	<0.001*	
Treatment	0.065	0.467	0.138	0.890	
Smooth predictors					
Coefficient	Estimate (*β*)	SEM	e.d.f	*F*‐value	*p*‐value
Angle of incidence (Yaw)	0.009	0.016	1.001	0.513	0.474
Time	−0.082	0.148	1.001	0.465	0.496
Time:Treatment	0.075	0.435	2.000	4.515	0.036*
Random predictors					
Coefficient	SEM				
Individual identity	1.260				

Note

Eye region temperature GAMM is weighted by relative angle of incidence to adjust for heteroskedasticity of temperature estimates across angles. Estimates (*β*) and standard errors (SEM) of smooth terms are averaged across knots. Asterisks (*) indicate significant effects. *N* = 8 individuals; *n* = 488 and *n* = 494 observations at the bill and eye region, resepctively. Deviance explained = 84.9% for GAMM‐predicting eye region temperature, and 63.5% for GAMM‐predicted bill temperature.

At the level of the bill, including angle of incidence as a coviariate in our model predicting mean surface temperature did not improve log‐likehood estimates (*X* ^2^ = −0.199, *p* = 0.990). Results of this adjusted model were similar to those from our model with angle of incidence excluded (Table 2; Figure 4b). Specifically, a significant interaction between treatment type and time was detected (*p *= 0.036; Table 2), with bill temperature of stress‐exposed individuals again declining after handling relative to control individuals (Figure 4b). Here, bill temperature averaged 1.4°C lower during handling than prior to handling in stress‐exposed individuals (pre‐handling: 32.5°C ± 0.470; post‐handling: 31.1°C ± 0.424), and again fell below that of control individuals after handling was initiated (stress‐exposed: 31.1°C ± 0.424; control: 31.6°C ± 0.528). Neither treatment type, time, nor angle of incidence significantly predicted bill temperature (*p*_treatment_ = 0.890; *p*_time_ = 0.496; *p*_yaw_ **= 0.474; Table 2).

### 3.3 Individual variation in stress‐induced thermal responses are detectable at the bill but not the eye region

**FIGURE 5:**
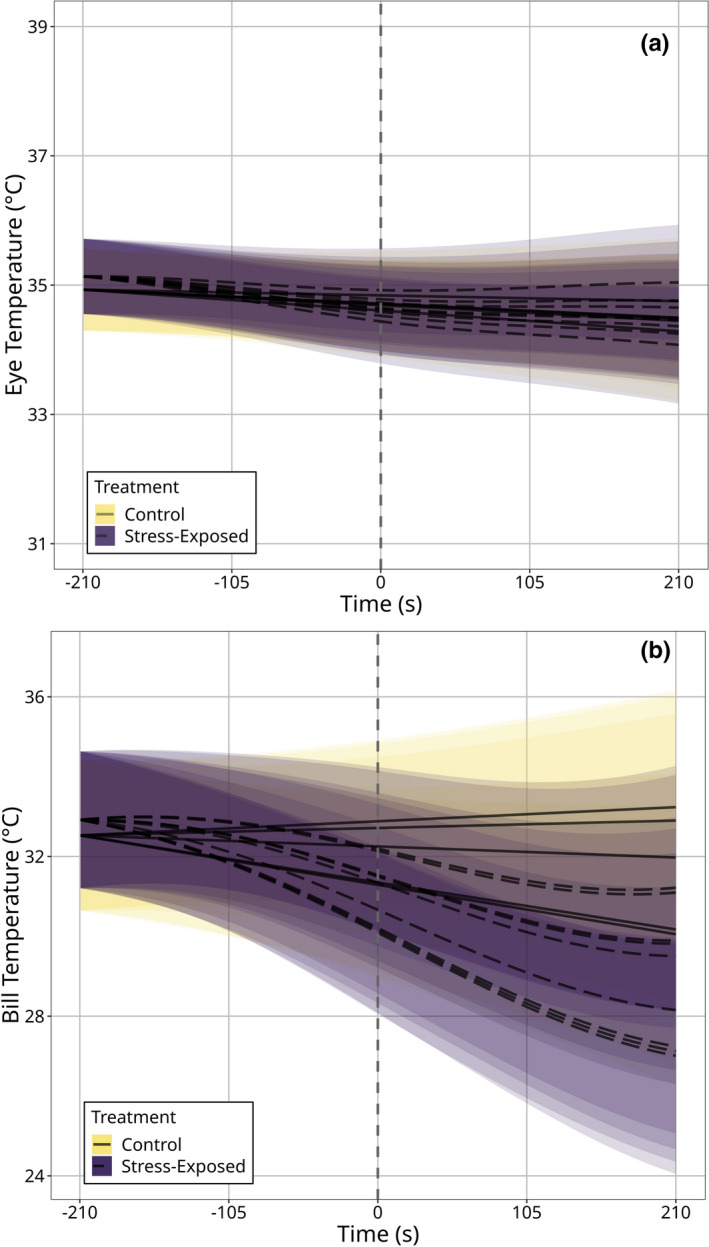
Surface temperature responses to acute stress exposure (handling) or control treatments across individuals. (a) Maximum eye region temperature (*n* = 9 individuals; *n*_control_ = 5, *n*_stress‐exposed_ = 8). (b) Maximum bill temperature (*n* = 10 individuals; *n*_control_ = 5, *n*_stress‐exposed_ = 9). Time "0" (indicated with a vertical dashed line) represents the time at which stress exposure treatments began. Each line represents the fitted response of a single individual to a given treatment type; solid lines represent control treatments and dashed lines represent acute stress exposure treatments. Trend‐lines are estimated from generalized additive mixed‐effects models (“GAMMs”) and are adjusted to account for differences in baseline surface temperature measurements among individuals. Ribbons represent 95% simultaneous confidence intervals around trends and are estimated per individual per treatment. Estimating individualized responses to treatment types significantly improved GAMMs predicting bill temperature (*p* < 0.001) but not eye region temperature (*p* = 0.082), according to chi‐squared difference tests

Inclusion of individual‐level responses to handling did not improve the explanatory capacity of our models for eye region temperature (*X* ^2^ = 9.822, *df* = 5, *p* = 0.082), but did improve the explanatory capacity of models for bill temperature (*X* ^2^ = 29.451, *df* = 8, *p* < 0.001), suggesting that individual differences in stress‐induced thermal responses were detectable at the bill alone (Figure 5).

## 4 DISCUSSION

Our results show that: (1) surface temperature responses to acute stress exposure were more prominent at the bill than at the eye region (Figure 3), and (2) individual differences in stress‐induced thermal responses were distinguishable at the bill alone (Figure 5). Interestingly, our results also show that changes in head orientation during thermographic imaging significantly influenced mean eye region temperature estimates (Figure S2A) with meaningful consequences on our capacity to detect and quantify stress‐induced thermal responses in birds (Table 2; Figure 4a). Notably, such effects of head orientation on surface temperature estimates appeared to be absent at the level of the bill in our study (Figure S2B), and the capacity to detect stress‐induced thermal responses at the bill was not contingent upon accounting for variation in head orientation among individuals (Table 2; Figure 4b). These findings suggest that while stress‐induced changes in eye region temperature may well approximate ANS‐mediate stress responsiveness in birds, both the relatively low magnitudes of thermal responses at this region, and their relatively high susceptibility to measurement error may hinder their practical utility (but see “Implications and recommendations for infrared thermographic studies” below). Conversely, the relatively large magnitudes of stress‐induced thermal responses at the bill, and the relatively low susceptibility to measurement error at this region suggest that those seeking to draw physiological inference from surface temperature measurements in birds may have greater success by focusing on the bill, rather than the eye region.

### 4.1 Regional differences in the magnitude of stress‐induced thermal responses

In our study, eye region temperature declined by an average of 0.4°C following exposure to a stressor (handling), after differences in head orientation among thermographic images were accounted for. While small, the magnitude of our observed decline is comparable to that reported for other domestic avian species (e.g., 0.4–0.6°C in domestic chickens, Edgar et al., 2013; Herborn et al., 2015; head angle categorized and accounted for in Herborn et al., 2015). Proximately, such a dampened thermal response to acute stress exposure at the eye region may be explained by a relatively limited ANS‐mediation of vascular flow to and from this area. In pigeons, ANS control over vascular motion has only been reported in nearby arterioles with low flow rates (i.e., choroidal vessels; flow rate = 227.65 mg/min/eye; Cuthbertson et al., 1997; Fitzgerald et al., 1990), where changes in blood flow in responses to vascular constriction are likely to be small and undetectable by external changes in temperature. Similar ANS control has yet to be described for the ophthalmic artery and facial vein in pigeons; however, given that pronounced constriction of either vessel may negatively affect ocular function (role of each vessel described in McDougal & Gamlin, 2011), it is unlikely that possible changes in flow‐rate would be sufficient to cause prominent differences in superficial temperature. Furthermore, the presence of local counter‐current exchangers (Midtgård, 1983, 1985) and possible vascular dilation at surrounding cephalic tissue (Nord & Folkow, 2019) may act to lessen temperature declines that accompany ANS‐mediated vascular construction at the eye region.

Contrasting the eye region, average declines in bill temperature during acute stress exposure reached 2.6°C when head orientation of individuals was not accounted for in our analysis, and 1.4°C when effects of head orientation of individuals (albeit nonsignificant) were corrected. Again, such declines closely reflected those reported for other avian species during a perceived challenge at sub‐thermoneutral temperatures (mean decline in bill temperature = 1.3°C among Great Tits during food restriction, Winder et al., 2020). Given both the lack of counter‐current exchangers in the bill (Tattersall et al., 2017), and the expectedly limited functional consequences of reducing vascular flow toward it (see Hagan & Heath, 1980; potential costs discussed in Winder et al., 2020), ANS‐control over vascular motion in the bill, and thus over bill temperature, is likely to be large. As such, an enlarged stress‐induced thermal response at the bill relative to the eye region is perhaps unsurprising.

Beyond regional differences in the degree of ANS control, regional differences in stress‐induced thermal responses may also be explained by broader, ultimate mechanisms driving their occurrence (i.e., by differentially shaping ANS‐mediated thermal responses across the body). For example, recent hypotheses suggest that stress‐induced changes in surface temperature may occur to reduce energetic expenditure toward thermoregulation when allocation of energy is required elsewhere (e.g., to support the stress response; see Lewden et al., 2017; Robertson et al., 2020a, 2020b); reviewed in Oka, 2018). Neuroanatomical observations have since supported this hypothesis by showing that limbic structures involved in activating the physiological stress responses may directly inhibit thermoregulatory processes at the level of the hypothalamus (reviewed in Angilletta et al., 2019). In birds, the bill is widely recognized as an important region of environmental heat exchange (Tattersall et al., 2017). Declines in bill temperature following exposure to a stressor may, therefore, occur to reduce heat loss to the environment, thereby reducing energetic expenditure toward thermoregulation when temperatures are below thermoneutrality (as in our study: ambient temperature =18°C; lower critical temperature = 22°C; Calder & Schmidt‐Nielsen, 1967). Supporting this hypothesis, observations of Great Tits in winter have shown that individuals may lower their bill temperatures beyond those typically observed in sub‐thermoneutral temperatures to reduced energetic costs associated with heat loss in challenging environments (there, food restriction), despite hypothesized costs to bill function (Winder et al., 2020). Alternatively, pronounced declines in bill but not eye temperature may merely reflect functional responses to minimize the risk of hemorrhage during exposure to an acute stressor (Darlington et al., 1986; McGuigan & Atkinson, 1921; discussed in Nord & Folkow, 2019; Jerem et al., 2015). In pigeons, the bill is often used as a weapon in defence of perceived threats (Ramirez & Delius, 1979), and as such, individuals may seek to divert blood away from the bill during threat perception for hemoprotective purposes. In any case, shaping of stress‐induced thermal responses by such ultimate processes is likely to bias the degree to which regional responses reflect broad‐scale, ANS activity among individuals. Without adequate control over environmental covariates (i.e., ambient temperature; a particular challenge in field studies) and a clear understanding of the mechanisms by which thermal response are shaped, disentangling such biases to seek inferences about individual stress‐physiology may remain tenuous.

### 4.2 Regional thermal responses to stress exposure among individuals

Previous studies in both mammals and birds have reported significant variation in the magnitude of stress‐induced thermal responses among individuals (Careau et al., 2012; Carere & Van Oers, 2004; Robertson et al., 2021). Our results support this finding, with regional thermal responses to handling (here, at the bill) differing significantly among Pigeons (Figure 5). Although the cause of such variation remains unclear, it is possible that the degree to which handling elicited a physiological stress response differed among our experimental individuals, with direct consequences on blood flow to, and temperature of, the bill. In Domestic Hens, the magnitudes of stress‐induced changes in wattle and comb temperature have been shown to vary according to stressor intensity, with stressors that contributed to higher levels of glucocorticoid secretion resulting in larger reductions in comb and wattle temperature than those that contributed to lower levels of glucocorticoid secretion (Herborn et al., 2015). Similarly, in House Sparrows, skin temperature was negatively correlated with the magnitude of glucocorticoid secretion following pharmacological stimulation of the HPA axis (Ouyang et al., 2021). Together, these findings strongly suggest that regional thermal responses to stress exposure may reveal useful information about individual variation in HPA axis, or ANS sensitivity.

As predicted, our results show that, unlike responses at the bill, the magnitude of stress‐induced thermal responses at the eye region are largely similar among individuals. This finding corroborates with recent observations that stress‐induced changes in eye region temperature of Domestic Hens remained similar across varying stressor intensities (Herborn et al., 2015). While the magnitude of stress‐induced thermal responses at the eye region may well be fixed among our experimental individuals (rationale discussed in Herborn et al., 2015), we suggest that an insufficient resolution to detect small variations in individual responses is a more probable explanation for our finding. During handling, eye region temperature declined by an average of 0.4°C, or between approximately 0.2°C and 0.6°C (95% confidence intervals), after correction for variations in head orientation. Such modest declines in surface temperature may be easily overshadowed by instrumental error (e.g., detector noise, drift, Playà‐Montmany & Tattersall, 2021; Minkina & Dudzik, 2009), or imaging biases (e.g., focus shifts, spot size variations, or changes in object orientation: Playà‐Montmany & Tattersall, 2021; Tattersall, 2016); reported here), leaving variation among individuals difficult to discern without careful and large‐scale repeated sampling. Our findings, therefore, suggest that individual differences in stress‐induced thermal responses at the eye region should be interpreted with caution until further research with careful control of common sources of error are conducted.

### 4.3 Effects of head orientation on regional estimates of surface temperature

Object orientation has been raised as a possible source of systematic measurement error in infrared thermographic studies (e.g., Herborn et al., 2015, 2018; Playà‐Montmany & Tattersall, 2021; Winder et al., 2020), yet to our knowledge, no studies have explicitly tested the effects of spatial orientation on surface temperature estimates in live animals. Our study, therefore, represents the first to do so. Indeed, previous studies seeking to account for individual orientation have sought to do so by either categorizing spatial orientation subjectively (Herborn et al., 2015, 2018) or by measuring the length of an anatomical structure within and across images (Winder et al., 2020; where the effects of a 3‐dimensional translation or 3‐dimensional rotation are inseparable). While these approaches are undoubtedly valuable for reducing measurement error attributed to variation in individual orientation, each probably lacks the capacity to robustly test the effects of object orientation alone on surface temperature estimates both within and across biological tissues.

Results of our study show that changes in individual orientation (here, the angle of incidence, or yaw, of the head) significantly influenced surface temperature estimates at the eye region, but not the bill. Intriguingly, changes in individual orientation did not appear to influence the uncertainty of surface temperature estimates at either tissue from our thermographic images. These findings are both corroborated by recent empirical findings, and yet to be reported. Specifically, using mounted samples of biological tissues, Playà‐Montmany and Tattersall (2021) showed that estimates of an object's emissivity significantly declined when rotated away from a thermographic sensor (i.e., when angle of incidence increased). As a consequence of such declines, surface temperatures of a given object were typically underestimated when the degree of rotation (or the angle of incidence) was large, similar to underestimation observed at the eye region here (Figure S2). Furthermore, Playà‐Montmany and Tattersall (2021) also reported that the effect of object rotation on surface temperature estimates varied across biological tissues types, with the degree of error fluctuating from ~2°C (fur from an American Mink, *Neovison vison*) to ~6°C (snake skin) when an angle of incidence approached 80°; we report a similar variation in the effects of object rotation on estimates of surface temperature among tissues (i.e., the highly karatinized bill, and soft eye region). Unlike Playà‐Montmany and Tattersall (2021), however, we show that failing to correct for the effects of object rotation, or orientation, on surface temperature estimates can bear meaningful consequences on the detection of physiological reponses to stimuli. Such a finding emphasizes the need to control for, or account for, changes in spatial orientation when seeking to draw inference from temperature values obtained by infrared thermography (raised in Playà‐Montmany & Tattersall, 2021; Winder et al., 2020).

### 4.4 Implications and recommendations for infrared thermographic studies

Our findings demonstrate that in some avian species, stress‐induced thermal responses at the bill may serve as more practical approximators of ANS‐responsiveness than those at the eye region. In Domestic Pigeons, stress‐induced changes in bill temperature appear more robust to concealment from systematic measurement error, and alone permit discrimination of individual responses to a stressor. Perhaps more critically, our results show that those wishing to quantify and interpret stress‐induced changes in surface temperature at any anatomical region would do well to either control for, or account for, differences in the orientation of individuals within infrared thermographic images. To this end, we report a robust and novel method that may be used by future researchers to estimate individual orientation in infrared thermographic images *a posteriori*. This method may be particularly valuable for those monitoring surface temperature at the eye region, or other anatomical regions with unknown thermal responses to changes in spatial orientation. We recognize, however, that estimating individual orientation using our proposed method may be tedious and time‐consuming, particularly when large numbers of thermographic images are to be used in analyses (the case for many laboratory‐based studies). Thus, those seeking to draw biological meaning from large numbers of thermographic images may prefer to simply control for variations in individual orientation by omitting images wherein the anatomical region of interest is not perpendicular to the lens of the thermographic device (as done in Winder et al., 2020). In field studies, however, collection of multiple images per study individual may be difficult, and the likelihood of the anatomical regions of interest falling perpendicular to the thermographic device may be low. In these cases, we advise researchers to estimate the orientation of individuals using the method proposed in this study.

## 5 CONCLUSION

Together, our study highlights the difficulty of inferring meaning about physiological state from noninvasive measurements of body temperature. Nevertheless, we provide evidence for the potential to do so, following careful consideration of sources of error and variation. In this study, we report a novel method that may be used to correct for one such source of error: variation in individual orientation. Although this method may be time‐intensive, it does afford researchers the opportunity to maximize inclusion of thermographic data, and it may yet be amenable to automation by machine learning. Overall, future studies would do well to investigate the implications of individual orientation on the capacity to mask correlations between physiological indicators of ANS responsiveness and regional surface temperature estimates.

## CONFLICT OF INTEREST

No competing interests are declared.

## AUTHOR CONTRIBUTIONS

JKRT, GFM, and GB conceived the study. JKRT executed the experiments, collected and analyzed the data, and wrote the first draft of the manuscript. GJT and OHW contributed to experiment execution. GFM, GB, OHW, and GJT contributed to manuscript revision.

## Supporting information



Figure S1

Figure S2

Supplementary Material

## Data Availability

All data and statistical code used in the construction of this study are available at https://github.com/joshuakrobertson/IR_Stress.
