# Supplementary figures and images for: Infrared thermography as a technique to measure physiological stress in birds: Body region and image angle matter
Source: Physiol Rep. 2021 May 31;9(11):e14865. doi: 10.14814/phy2.14865 (PMC8165734; doi:10.14814/phy2.14865)

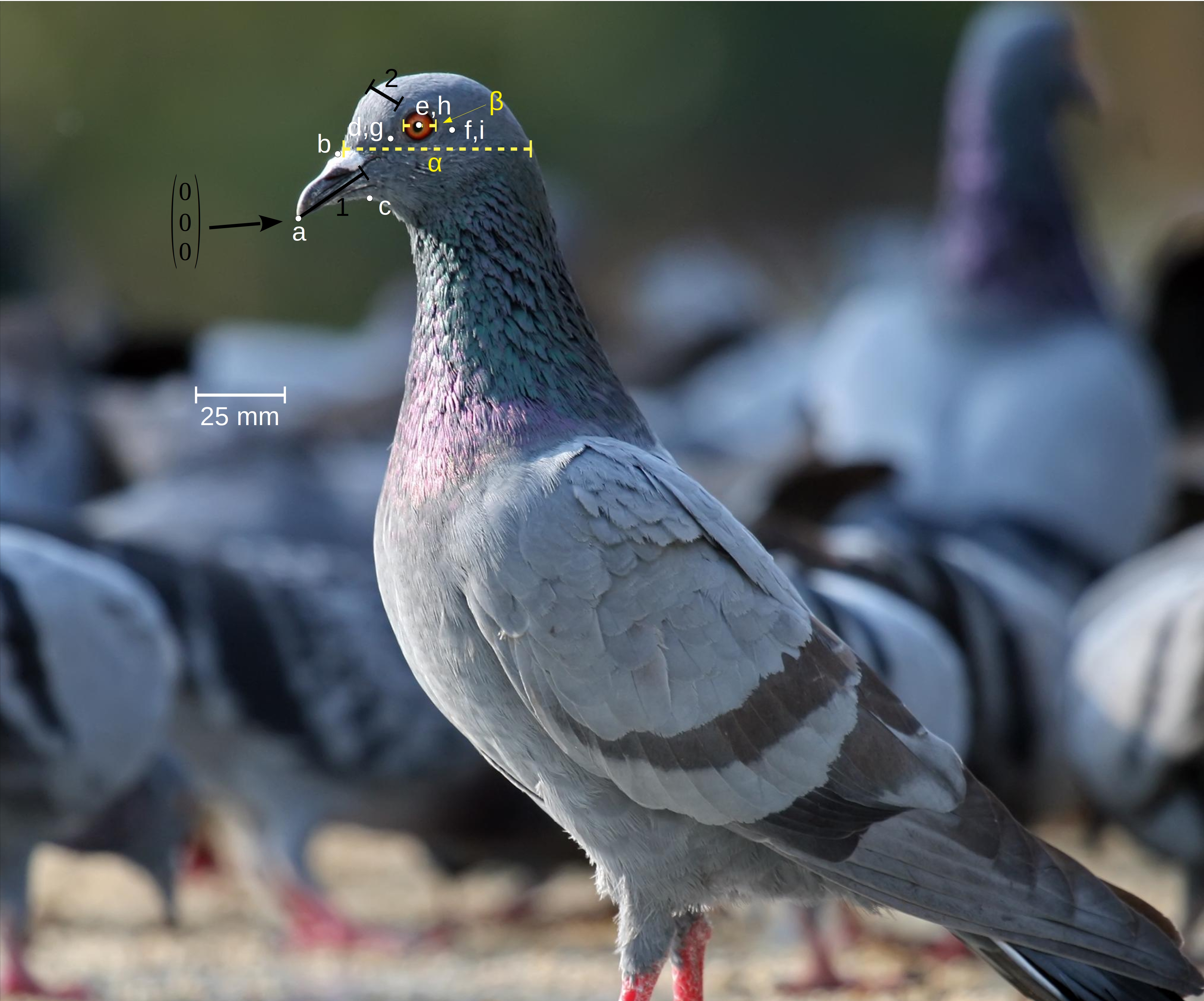

Supplement: Supplementary file 1 — Figure S1 [file PHY2-9-e14865-s003.jpg]

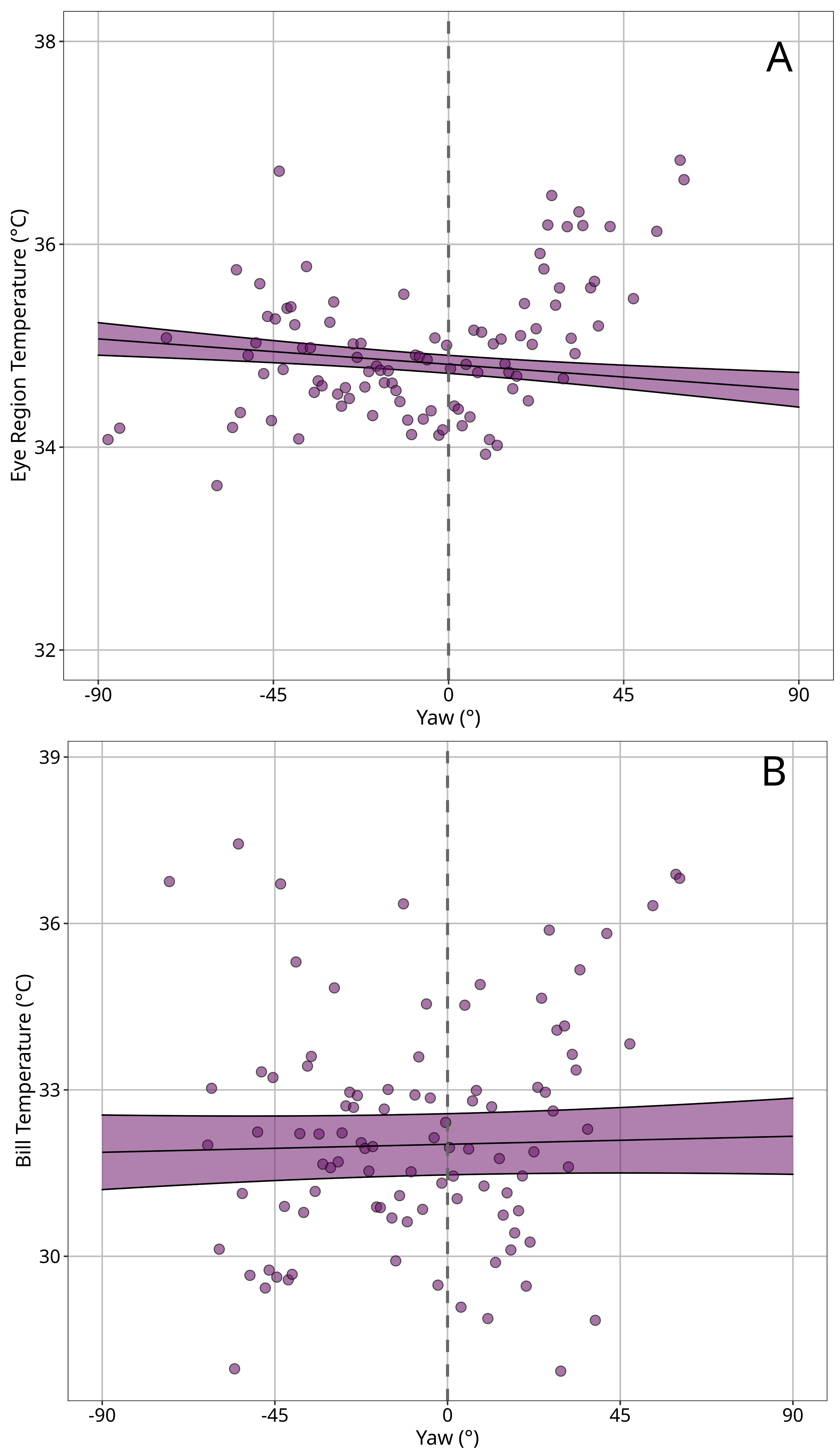

Supplement: Supplementary file 2 — Figure S2 [file PHY2-9-e14865-s001.jpg]
