# Supplementary material for: Infrared thermography as a technique to measure physiological stress in birds: Body region and image angle matter
Source: Physiol Rep. 2021 May 31;9(11):e14865. doi: 10.14814/phy2.14865 (PMC8165734; doi:10.14814/phy2.14865)
Supplement: Supplementary file 3 — Supplementary Material [file PHY2-9-e14865-s002.docx]

**Supplemental Information**

# Tables

**Supplemental Table 1: Influence of stress exposure on facial temperature of a randomly-sampled subset of domestic pigeons, prior to correction for head orientation; results of two GAMMs.** Estimates (β) and standard errors (s.e.m.) of smooth terms are averaged across knots. Asterisks (*) indicate significant effects. N_individuals_ = 7; n_observations_ = 511 at the eye region and n._observations_ = 502 at the bill. Deviance explained = 82.1% for GAMM predicting eye region temperature, and 65.2% for GAMM predicted bill temperature.

| Eye Region Temperature | | | | | |
| --- | --- | --- | --- | --- | --- |
| Parametric Predictors | | | | | |
| Coefficient | Estimate (*β*) | s.e.m. | *t*-value | *p*-value |  |
| Intercept | 34.677 | 0.350 | 98.990 | <0.0001* |  |
| Treatment | -0.005 | 0.495 | -0.010 | 0.992 |  |
| Smooth Predictors | | | | | |
| Coefficient | Estimate (*β*) | s.e.m. | e.d.f | *F*-value | *p*-value |
| Time | -0.059 | 0.036 | 1.000 | 6.245 | 0.013* |
| Time:Treatment | 0.031 | 0.083 | 0.732 | 1.919 | 0.103 |
| Random Predictors | | | | | |
| Coefficient | s.e.m. |  | | | |
| Individual Identity | 0.523 |  |  |  |  |
| Bill Temperature | | | | | |
| Parametric Predictors | | | | | |
| Coefficient | Estimate (*β*) | s.e.m. | *t*-value | *p*-value |  |
| Intercept | 31.787 | 0.881 | 36.062 | <0.0001* |  |
| Treatment | -1.220 | 1.242 | -0.982 | 0.327 |  |
| Smooth Predictors | | | | | |
| Coefficient | Estimate (*β*) | s.e.m. | e.d.f | *F*-value | *p*-value |
| Time | 0.006 | 0.154 | 1.001 | 0.002 | 0.970 |
| Time:Treatment | 0.311 | 0.442 | 1.684 | 7.476 | 0.001* |
| Random Predictors | | | | | |
| Coefficient | s.e.m. |  | | | |
| Individual Identity | 1.380 |  |  |  |  |

# Figures

**Supplemental Figure 1 | Standardized digital image of a Domestic Pigeon used to predict 3-dimensional orientation of study individuals in infrared thermographic images.** White dots represent standardized and visible landmark positions and solid black lines with numeric labels represent distance measurements with known lengths in the world co-ordinate system (“w.c.s.”). Line (1) used to calibrate scaling between pixels and length in w.c.s.; line (2) used to estimate distance between landmarks on the left and right side of head. Dashed yellow lines with Greek labels represent distance measurements with known lengths in the world co-ordinate system used to validate scaling estimates derived from black lines. Bill tip was set the w.c.s. origin (point 0,0,0 [x,y,z]). Latin letters label individual landmarks; landmarks a-f are visible and landmarks g-i are not. (a) bill tip: w.c.s. position (x,y,z) = (0,0,0). (b) upper caudal cyr: w.c.s. position = (13.439, 0, 18.816). (c) lower caudal mandible: w.c.s. position = (19.991, 0, 5.712). (d) left lower rostral periorbital area: w.c.s. position = (26.543, 9.700, 20.496). (e) center of left eyeball: w.c.s. position = (34.439, 12.200, 26.040). (f) left lower caudal periorbital area: w.c.s. position = (42.671, 9.700, 25.032). (g) right lower rostral periorbital area: w.c.s. position = (26.543, -9.700, 20.496). (h) center of right eyeball: w.c.s. position = (34.439, -12.200, 26.040). (i) right lower caudal periorbital area: w.c.s. position = (42.671, -9.700, 25.032). Length of (1) in w.c.s. = 22.060 mm (equal to marginal mean of premaxilla length across males and females; Johnston [1990]). Length of (2) in w.c.s. = 19.400 mm (equal to mean greater skull width of males; Johnston [1990]; range of greater skull width across males and females = 19.010 mm – 21.390 mm; Goldberg [1999]). Length of (α) in w.c.s. = 52.239 mm (range of greater skull length; 46.420 mm – 55.090 mm; Goldberg [1999]). Length of (β) in w.c.s. = 8.619 mm (mean anterior lens diameter ± s.d. = 8.248 ± 2.226 mm; Donovan [1978]). Unmarked image captured by Muhammad Mahdi Karim and obtained under GFD.12 licence from Wikimedia Commons.

**Supplemental Figure 2 | Effect of head angle (relative yaw) on surface temperature estimates drawn from infrared thermographic images of Domestic Pigeons.** Trends at the eye region **(A)** and bill **(B)**. A yaw of -90° represents the bill oriented directly toward the thermographic camera while a yaw of 90° represents the bill orientated directly away from the thermographic camera. Dots represent average surface temperature values for a given degree of yaw, and trend-lines represent marginal means and are estimated from generalized linear models including time, experimental treatment type, and an interaction between each as fixed predictors. Ribbons represent 95% Wald confidence intervals around trends. Eye region temperature, but not bill region temperature significantly declined across yaw (*p_eye_* < 0.001; *p_bill_* = 0.474).
